# Nongenetic Photostimulation of hiPSC Neurons Using Plasmonic Nanopyramids

**DOI:** 10.1021/acsphotonics.5c01708

**Published:** 2025-10-27

**Authors:** Rustamzhon Melikov, Giuseppina Iachetta, Marzia Iarossi, Marta d’Amora, Christian Tentellino, Julien Maxime Hurtaud, Francesco Tantussi, Michele Dipalo, Francesco De Angelis

**Affiliations:** † Italian Institute of Technology, Genoa 16163, Italy; ‡ Department of Biomedical Engineering, Technion - Israel Institute of Technology, 32000 Haifa, Israel; § Department of Biology, University of Pisa, 56127 Pisa, Italy

**Keywords:** electrophysiology, photostimulation, microelectrode arrays, plasmonics, locilized surface plasmon resonance, neurons, human induced pluripotent stem cell, capacitive photocurrent

## Abstract

Human-induced pluripotent stem cell (hiPSC)–derived neurons offer a powerful platform for replicating key aspects of human neurodevelopment, synaptic connectivity, and ion channel expression. However, their electrophysiological investigation remains challenging, particularly for studies aiming to elicit neuronal activity with minimal perturbation of physiological conditions. In this study, we integrated plasmonic gold pyramids onto commercial titanium nitride (TiN) microelectrode arrays (MEAs). The presence of these plasmonic structures enhanced the generated photocurrent by more than 30-fold and simultaneously reduced the electrode impedance by approximately 6-fold. Leveraging the unique optical properties of plasmonic nanostructures, we demonstrate that gold pyramids enable efficient neuronal photostimulation at low light intensities with minimal perturbations. Our approach combines nongenetic optical stimulation with high-resolution electrophysiological recording, providing precise spatiotemporal control of neuronal activity. These advances highlight the potential of our plasmonically enhanced MEA technology to bridge the gap between preclinical research and human neurological applications.

## Graphical abstract

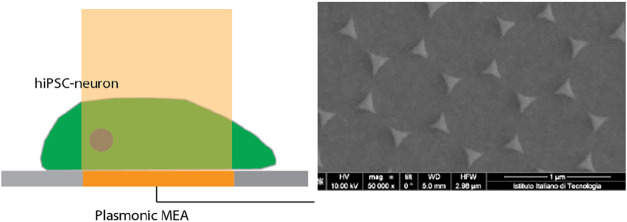

## Introduction

Human induced pluripotent stem cell (hiPSC)–derived neurons overcome many of the challenges faced by rodent neurons^1–2,3^ by providing a model system that recapitulates key aspects of human neurobiology. This emerging neuronal model capture the genetic and molecular landscape of human neurons, thereby offering higher predictive power for studying neurotoxicity, neuropharmacology, and the mechanisms underlying neurodegenerative disorders.^4,5^ By enabling the generation of patient-specific neuronal populations, hiPSC technology opens the door to personalized medicine approaches, reduces the high failure rates observed in clinical trials, and allows researchers to probe the subtleties of human neuronal network dynamics that cannot be mimicked by rodent neurons. The quest to unravel the complexities of the human brain and its intricate neural networks has long been a central focus of neuroscience research. Extracellular signals are essential for interacting with biological systems and are key to restoring lost functionalities, understanding cellular mechanisms, and regulating neural networks.^6,7^ Light provides a noninvasive method to modulate brain activity with high spatial and temporal precision. While optogenetics involves introducing light-sensitive opsins through viral means, concerns regarding genetic modifications currently limit its clinical application.^8–10^ On the other hand, optical stimulation shows promise in controlling neural activity without genetic intervention. Among the various tools and techniques available to neuroscientists, photostimulation combined with microelectrode arrays (MEAs) has emerged as a powerful and versatile method.^11^ This innovative approach combines the precision of light-based control with the spatial and temporal resolution of microelectrodes, allowing for the detailed exploration of human neuronal activity.^12^ Photostimulation on MEA enables researchers to investigate the spatiotemporal neuronal response with unmatched sensitivity. When coupled with photostimulation, this technology enables precise control over neuronal activity in specific regions, adding a dimension of manipulation to the observation.^11^ This powerful combination allows not only monitoring but also controlling the activity of human neurons, thus promising valuable insights into the neural basis of cognition and behavior.^13,14^ The potential applications are vast, ranging from the study of healthy brain function to investigations of neurological disorders, offering new avenues for understanding, diagnosis, and therapeutic strategies.^15–17^

Recent advances in plasmonics have further enriched this landscape. Plasmonic nanostructures enable the focusing of incoming radiation into a smaller spatial profile than the incident light, a phenomenon achieved through localized surface plasmon resonance (LSPR).^18–24^ Moreover, the energy from light-induced plasmons can transfer to the nanostructure’s conduction band, generating highly energetic electrons called “hot electrons.” These electrical currents can be harnessed and directed to stimulating neurons through controlled materials. However, despite considerable potential for cell stimulation, the application of plasmonics has primarily focused on modulating neurons via photothermal effects. Namely, light is converted to heat energy due to the damping of plasmon oscillations. The resulting temperature changes prompt alterations in membrane potential by inducing a transient shift in membrane capacitance.^25^ Recent studies have also suggested that decorating metals could potentially enhance capacitive currents, contributing to further advancements in this field.^25^ Photocapacitive stimulation of neurons is considered a safe method because it relies on transient, noninvasive, and purely capacitive perturbations of the cell membrane potential. This is in contrast to faradaic currents, which are associated with electrochemical reactions that can alter or even damage the cell. Therefore, capacitive stimulation is preferred over direct current injection or photothermal stimulation, as it presents a minimal risk of tissue injury while providing precise and reversible control of neuronal activity.

In this study, we harness these advances by fabricating plasmonic gold pyramids on commercial titanium nitride MEA and culturing hiPSC-derived glutaneurons on them. This plasmonically enhanced MEA platform exhibits an impedance up to 6 times lower than that of unmodified electrodes at 1 kHz. The capacitive photocurrents are at least 30 times higher, enabling reliable photostimulation while reducing light intensities by a factor of 2.6. This minimizes photodamage while maintaining precise control of neuronal firing.^27^ Moreover, photocapacitive behavior of the electrode contributes to safe photostimulation for neurons in contrast with stimulation based on faradaic currents. The integration of hiPSC-derived neurons into our system not only provides a more physiologically relevant model for human brain function but also facilitates a deeper understanding of the cellular and network-level mechanisms that underlie neurological disorders. To enable access to a wider community, we employed commercial titanium nitride (TiN) MEAs provided by multichannel Systems. Consequently, this approach promises to advance both fundamental neuroscience and the development of novel and clinically translatable neurotherapeutic strategies.

## Results

### Characterization of Au-Pyramid MEA

MCS-MEAs (multichannel systems-microelectrode arrays) are a widely utilized platform in electrophysiology laboratories due to their numerous technical advantages, including high-quality extracellular recordings, versatility in application, and mechanical robustness. Typically, these systems employ titanium nitride (TiN) electrodes, which are favored for their low impedance and durability.

In this study, microelectrode arrays (MEAs) with titanium nitride (TiN) electrodes commercially available from multichannel systems (MCS) were selected as the substrate for the fabrication of gold nanopyramids and used as control devices. These MCS-MEAs comprise 60 individual TiN electrodes, each with a diameter of 30 μm and separated by an interelectrode distance of 200 μm. The surface of the TiN electrodes exhibits a porous architecture with nanoscale roughness, accompanied by interspersed nanogaps between TiN protrusions, which promote better adhesion for hiPSC neurons. Figure 1a shows a schematic of the underlying concept for photostimulation of hiPSC neurons using microelectrode arrays (MEAs). In Figure 2b, the SEM image of the Au pyramid is shown.

**Figure 1:**
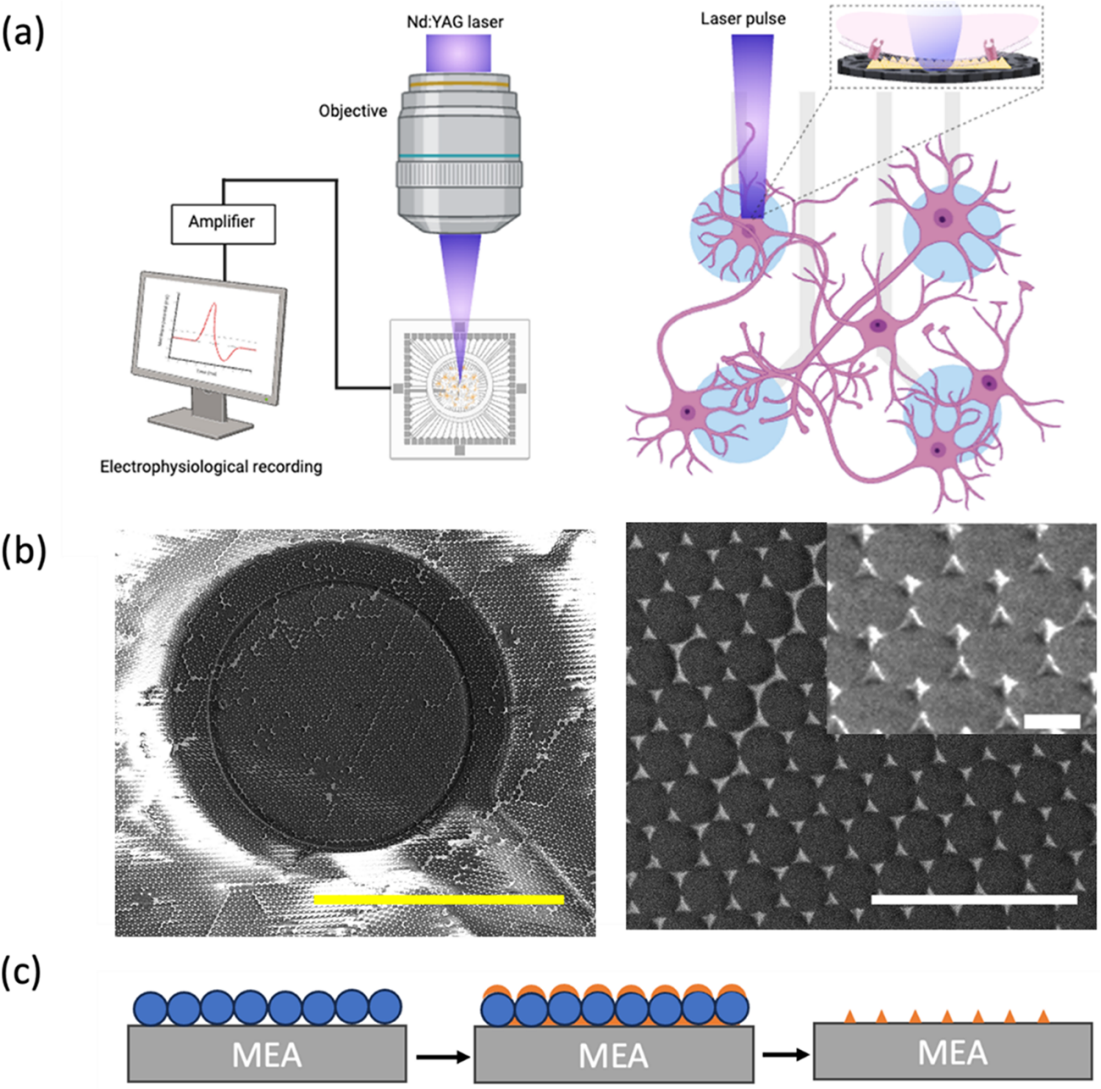
(a) Schematic of optoelectronic photo stimulation and recording setup. (b) SEM images of Au pyramids. Left: the whole electrode; scale bar, 30 μm. Right: zoom, top view, and tilted (inset) scale bars, 3 μm and 500 nm. (c) Scheme of the fabrication of Au pyramids using the liquid lithography method on a control MEA.

**Figure 2:**
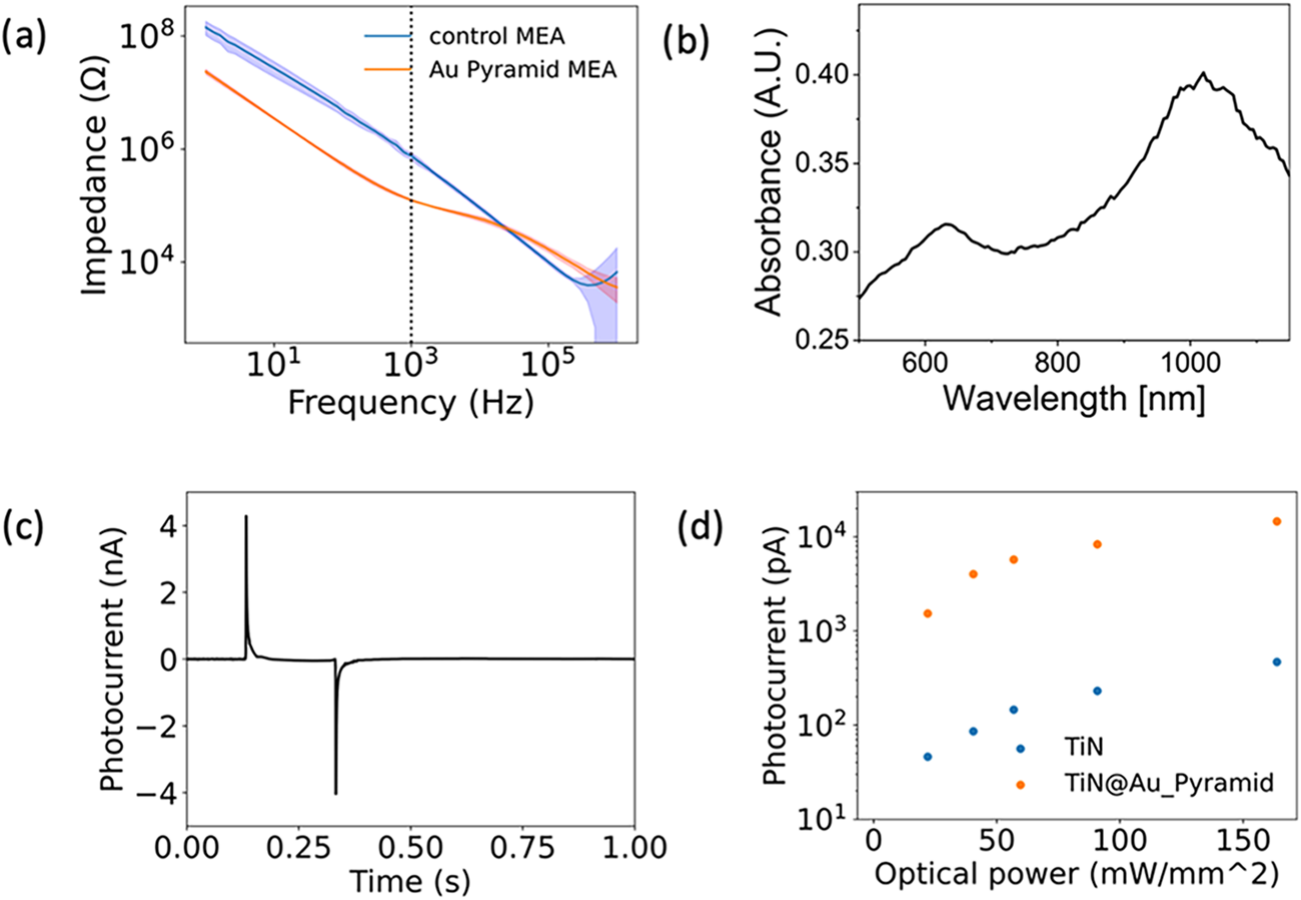
(a) Electrochemical impedance spectroscopy of control MEA and Au-pyramid MEA. (b) Absorbance of Au pyramids. (c) Photocurrent generated by Au pyramids on a control MEA electrode. (d) Comparison of photocurrent generated by control MEA and Au-pyramid MEA.

As detailed in Figure 1c, gold nanopyramids were integrated onto the TiN electrodes via liquid lithography. A gold thickness of 200 nm was selected so to ensure that the Au nanopyramids exhibit a plasmonic resonance peak at 1064 nm, optimizing the photostimulation process.^24^ Additionally, the presence of the Au nanopyramids further enhances neuron adhesion, augmenting the already rough TiN surface and facilitating better interaction with the neurons.^26^ In Figure 1b, the structural details of the Au nanopyramids are shown using scanning electron microscopy (SEM) and Au pyramids on the TiN electrode shown in Figure S1, while Figure 1a presents the schematic representation of the photostimulation and recording setup.

We conducted electrochemical impedance spectroscopy on both the control MEAs and the Au-pyramid MEAs, the latter of which exhibits plasmonic properties. As shown in Figure 2a, the impedance of the Au-pyramid MEAs is noticeably lower compared to that of the control MEAs. After three cultures, we did not observe significant change in EIS of Au pyramid on the TiN electrode (Figure S2). This reduction in impedance can be attributed to the presence of the gold pyramids, which enhance the electrode’s conductivity. As a result, the lower impedance is expected to improve the recording of hiPSC neuronal signals by increasing the signal-to-noise ratio in comparison to the control MEAs, potentially leading to more accurate and high-fidelity recordings of neuronal activity.^27^

To further validate these findings, we used absorption spectroscopy to reveal the plasmonic peak of the Au pyramids, which corresponded to 1064 nm (Figure 2b). Following this, we examined the response of the plasmonic Au-pyramid MEAs to a 1064 nm laser. Under laser illumination, capacitive photocurrent was generated, which is advantageous compared to faradaic photocurrent for neuron stimulation,^28^ as it minimizes potential damage to electrodes and surrounding tissue by avoiding chemical and structural changes during the stimulation pulse.^29^ This indicates that the photostimulation of plasmonic systems is potentially safer and more effective for use with hiPSC neurons as it generates the desired photocurrent without potentially harmful faradaic reactions.^30^

Furthermore, the photocurrent produced by the plasmonic Au-pyramid MEAs was found to be more than a factor of 30 higher than that generated by the control MEAs. This significant increase in the photocurrent highlights the crucial role of Au pyramids in enhancing the plasmonic response and facilitating effective neuronal photostimulation. By leveraging the unique properties of plasmonic nanostructures, we demonstrate that these gold nanopyramids enable effective photostimulation of hiPSC neurons at low light intensity levels.

### Photostimulation of hiPSC Neurons

Photostimulation was achieved by focusing an ultrafast pulsed 1064 nm laser at the interface between the neurons and the plasmonic Au-pyramid MEA, as well as the control MEA. The laser was applied with pulse trains lasting between 20 ms to 30 s and an average power output ranging from 2 to 10 mW. *We separate two temporal regimes of photostimulation: (i) concurrent, on-beam responses during illumination (* Figure 3 *) and (ii) postillumination network responses after the light is OFF (* Figure 4 *)*.

**Figure 3:**
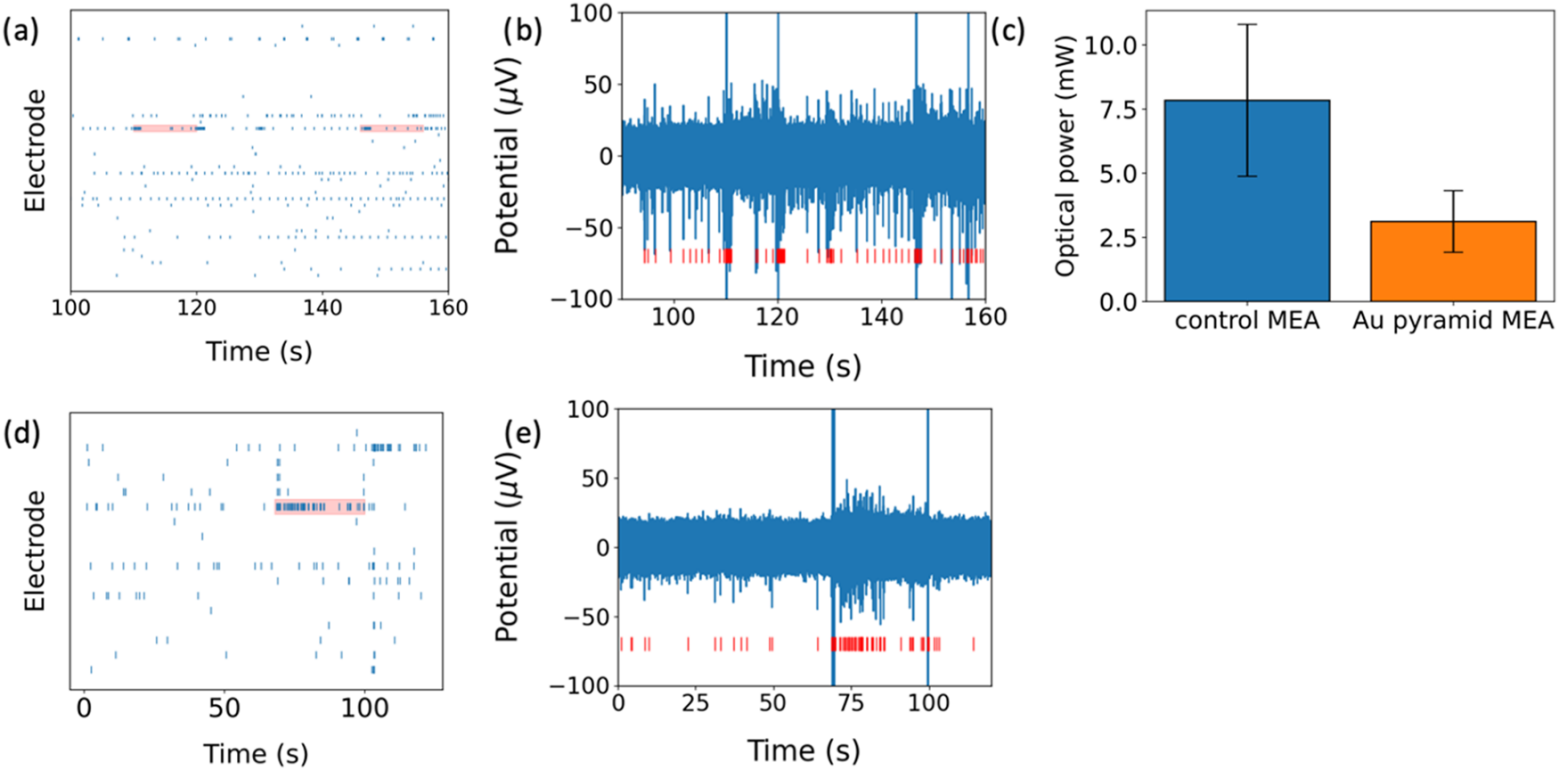
(a) Raster plot of representative hiPSC neurons on control MEA during illumination (laser ON). Red blocks show 1064 nm laser illumination at 8.65 mW optical power. (b) Illuminated electrode, neurons fire more action potentials under 1064 nm laser illumination. (c) Photostimulation threshold of control MEA and Au-pyramid MEA. *Summary across cultures (n = 3, 10 electrodes)*; *overall reduction =* **2.6** *×*. (d) Raster plot of representative hiPSC neurons on Au-pyramid MEA during illumination (laser ON). Red blocks show 1064 nm laser illumination at 2.23 mW optical power. (e) Illuminated electrode, neurons fire more action potentials under 1064 nm laser illumination.

**Figure 4:**
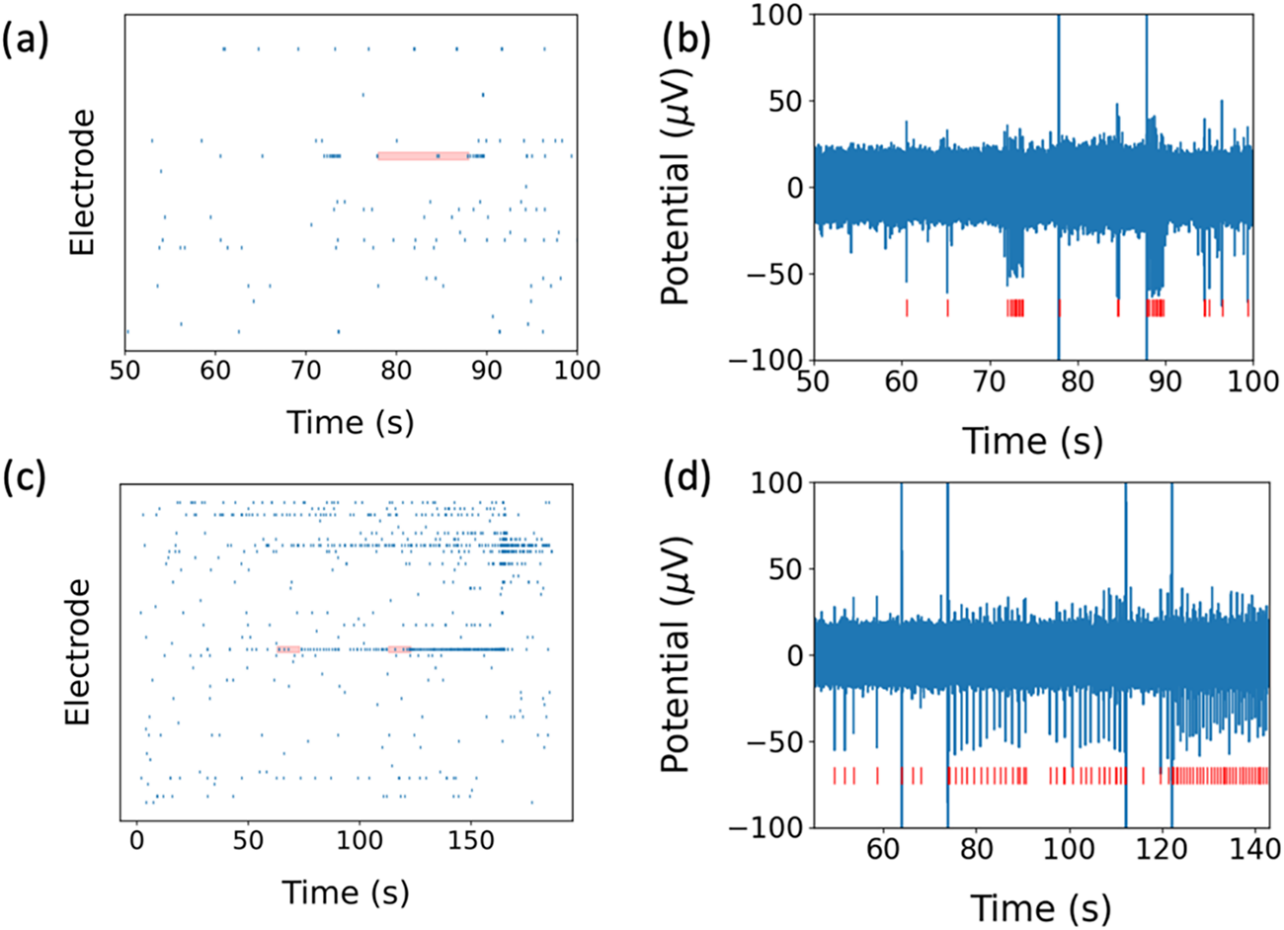
(a) Raster plot of representative hiPSC neurons on control MEA during post illumination (laser OFF). The red block shows 1064 nm laser illumination at 8.65 mW optical power. (b) Illuminated electrode, firing rate is increased immediately after 1064 nm laser illumination. (c) Raster plot of hiPSC neurons on Au-pyramid MEA during post illumination (laser OFF). The red block shows 1064 nm laser illumination at 2.23 mW optical power. (d) Illuminated electrodes; neurons produce more action potential after 1064 nm laser illumination.

*During illumination (laser ON)*, *the illuminated electrode exhibits a spike-rate increase confined to the stimulus window (* Figure 3 *a–c)*. As demonstrated in the raster plot (Figure 3a), hiPSC neurons cultured on control MEAs exhibited an increase in electrical activity upon exposure to a 1064 nm laser illumination. at 8.65 mW optical power and 10 s pulse duration. Figure 3b quantifies this observation by showing a significant increase in the spike rate of the hiPSC neurons. Similarly, as presented in the raster plot (Figure 3d), hiPSC neurons on plasmonic Au-pyramid MEAs also showed an increase in electrical activity under lower optical power at 2.23 mW and a 10 s pulse duration. Figure 3e further confirms this by displaying an increased spiking rate in these neurons.

In a representative culture (Figure 3a,b), Au-pyramid MEAs lowered the optical threshold by 3.8× relative to TiN. Across three cultures and ten electrodes, the mean reduction was 2.6× (Figure 3c). While both MEA configurations effectively demonstrated neuronal photostimulation, the plasmonic Au-pyramid MEAs required a lower optical power threshold to achieve neuron stimulation, highlighting the enhanced efficiency of the Au pyramids in facilitating photostimulation (Figure 3c).

After illumination (laser OFF), we observe a spike rate increase (Figure 4a–d). As depicted in the raster plot, we observed an increased neuronal spike rate in stimulated electrodes immediately after laser illumination. Specifically, hiPSC neurons cultured on the control MEA exhibited a marked increase in electrical activity after exposure to the 1064 nm laser. This increase is further illustrated in Figure 3b, which confirms a higher spike rate in these hiPSC neurons postillumination. Similarly, the raster plot shows that hiPSC neurons on the plasmonic Au-pyramid MEA also demonstrated heightened electrical activity following 1064 nm laser stimulation. This spike rate increase is quantified in Figure 3e, providing further evidence of the neuronal response to laser stimulation.

Both MEA configurations effectively facilitated neuronal photostimulation; however, plasmonic Au-pyramid MEA required a lower optical power threshold to induce neuronal activity, demonstrating enhanced efficiency compared to the control MEA. This finding highlights the advantages of the Au-pyramid-modified electrodes, which enabled more effective and precise photostimulation of the cultured neurons.

Following consecutive photostimulation attempts, the effects of photostimulation persisted briefly after laser exposure. However, once laser illumination ceased, the spike frequency in both the control MEA (Figure 5a) and the plasmonic Au-pyramid MEA (Figure 5b) gradually returned to the baseline levels (Figure 5). This observation suggests that the photostimulation induced a reversible neuronal response that was temporary, with the neurons returning to their normal firing patterns after the cessation of the laser illumination. The observed effect indicated that photostimulation induces a reversible and nonpermanent modulation of neuronal activity. Such transient neuromodulation is particularly advantageous for research or therapeutic approaches requiring precise temporal control without cumulative adverse effects. The transient nature of the photostimulation effect suggests potential uses in real-time neural network manipulation and promising applications in noninvasive neuromodulation.

**Figure 5:**
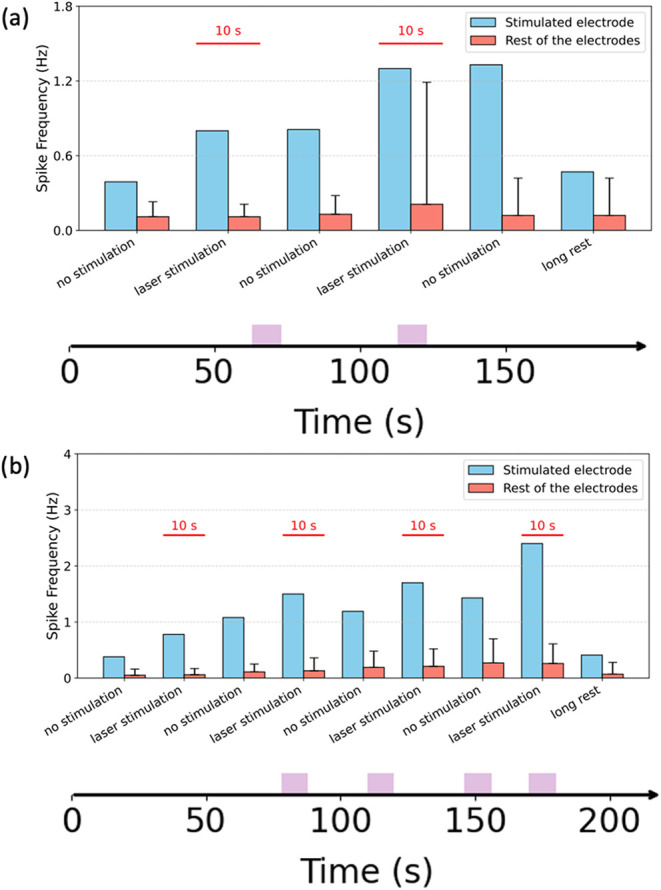
(a) Spike frequency under rest and illumination conditions of hiPSC neurons on control MEA. Blue bars show 1064 nm laser illumination. Orange bars show average spike frequency of hiPSC neurons on control MEA. *X* axis at the bottom indicates laser illumination time. (b) Spike frequency under rest and illumination condition of hiPSC neurons on Au-pyramid MEA. Blue bars show 1064 nm laser illumination. Orange bars show average spike frequency of hiPSC neurons on Au-pyramid MEA. *X* axis at the bottom indicates laser illumination time. Photostimulation consisted of 10 s illumination epochs at 1064 nm, separated by ≥30 s OFF intervals (no exposure). Protocols included repeated ON–OFF cycles followed by a long rest of 60 s OFF. Exact ON/OFF timings are annotated on the time axis of Figure 5.

The reader may notice that in Figure 5, the measurements of the spike frequency on the stimulated electrode have no error bars. Indeed, it is possible to estimate the error for a single electrode by calculating the variance of the spike frequency along the time. However, this would be misleading. In fact, error bars derived from a single electrode would reflect the temporal variability of spike frequency, whereas those calculated across multiple electrodes would instead reflect spatial variability among electrodes within the same time window. For this reason, we reported a single (stimulated) electrode with no error bars. To ensure robustness, we performed experiments on three independent cell cultures and obtained comparable results, which were not reported in the manuscript for brevity.

## Discussion and Conclusion

In this study, we successfully demonstrated the fabrication and application of plasmonic gold pyramids on commercial control MEAs to enhance photostimulation of hiPSC-derived neurons compared to the control MEAs. Our findings indicate that the integration of these plasmonic nanostructures significantly improves neuronal stimulation efficiency while minimizing the required optical power of about 3 times. This value may not appear very high, but several key aspects must be considered. First, the plasmonic system is not optimized. As shown in Figure 2B, the optical absorbance of the pyramid array at 1064 nm is about one-third. This is a remarkable value, given that the filling factor of the pyramids is only 10% in area, thus leaving good margins from improvements. The measured enhancement factor of 2.6× therefore reflects only partial light coupling. With optimized optical coupling, the factor could plausibly reach around 5, which would already represent a remarkable improvement, especially relevant for neurons, where excessive light exposure can cause overheating or cell damage. Second, it is worth noticing that the pyramids primarily enhance photocapacitive effects. Their impact on the cell membrane potential largely depends on the interface area between the pyramid and the cell membrane. As discussed, the filling factor of the pyramids is only 10%, hence increasing the filling factor or optimizing the nanostructure geometry could improve both pyramid-cell coupling and photocurrent, yielding a double performance gain.

Overall, these considerations indicate that electrode geometry and the cell–electrode interface play a dominant role compared with purely optical or electrical effects. In fact, although the photocurrent increases by a remarkable 30× factor, the corresponding spike frequency gain is roughly 10× lower, thus leaving a good margin for improvements.

In our system, hiPSC neurons on Au-pyramid MEAs reach a threshold at ∼2 to 10 mW average power at 1064 nm, with 2.6× mean across three cultures (Figure 3). In comparison, Organic photocapacitor devices (OEPCs) achieve capacitive stimulation with millisecond-scale pulses at ∼13 mW mm^–2^. However, they are flat capacitors working in the visible range of light (660 nm), which are hardly usable in the NIR range that is safer for cell culture. Plasmonic metamaterial interfaces have likewise demonstrated label-free NIR stimulation on MEAs with ultrafast pulses, validating the all-optical, nongenetic approach at the network level, potentially combinable with thermal therapies.^23,31^ Gold-nanoparticle strategies enable highly localized photothermal stimulation but require biochemical functionalization and introduce additional translational constraints.^32^

Beyond threshold, our Au-pyramid electrodes lower impedance and increase capacitive photocurrent, which together widen SNR and operating regime at reduced optical power (Figure 2). These trends are consistent with reports that advanced electrode coatings (e.g., PEDOT-based composites, graphene) improve charge transfer and recording fidelity; our platform combines such electrical benefits with an integrated photocapacitive actuation pathway on a standard MEA substrate.^33^

Because dose scales ∼linearly with average power at fixed pulse timing, our 2.6× mean threshold reduction materially lowers the photothermal and photochemical response of the electrode while preserving efficacy. Coupled with lower impedance and larger photocurrents, this creates a practical approach for (i) long, repeated protocols on human iPSC networks with reduced photodose, (ii) closed-loop, electrode-addressable perturbations during simultaneous recording, and (iii) high-throughput pharmacology and disease-model studies where each site can serve as a reproducible, label-free actuator.

Future research directions include optimizing plasmonic nanostructure design for specific wavelengths, investigating long-term neuronal responses, and expanding the potential applications of this technology in clinical and therapeutic settings. Ultimately, our work highlights the promising role of plasmonically enhanced MEA platforms in advancing human neuroscience and neuro-engineering.

## Methods

### Fabrication of Au Pyramids

The fabrication route to the Au NP arrays is based on colloidal lithography. First, negatively charged polystyrene (PS) spheres (purchased from microParticles GmbH) with superficial carboxylic acid groups and an average diameter of 750 nm were assembled in a hexagonally close-packed (hcp) monolayer. This was achieved by using an interfacial air–water self-assembly technique.^34,35^ Briefly, the aqueous solution of PS spheres (5 wt %) was diluted in a 1:1 ratio in ethanol and then pipetted onto a Si wafer suspended at an angle of 80◦ with its bottom edge in contact with a water bath. The Si wafer was first treated with an oxygen (O2) plasma cleaner (power: 100 W, flow rate: 25 sccm; Gambetti Plasma Cleaner System) for 10 min to make the surface completely hydrophilic to encourage spreading. In this way, the excess PS spheres could flow away in the water bath while ordered hcp monolayers were formed on the Si water after the solvent evaporates. A second plastic beaker was filled with deionized water, and the pH was increased up to 9 by adding a solution of sodium hydroxide. The pH of the water bath played an important role in the subsequent step in which the Si wafer was gently dropped in the water bath to transfer the PS spheres’ monolayers at the water–air interface of the second bath. The pH of the water bath affected the repulsion between neighboring PS spheres because at higher pH values, the surface charge density on each particle increased as a result of the deprotonation of the carboxylic acid groups.^36^ By increasing the particles’ electrostatic repulsion, the length of the triangular interstices formed by neighboring spheres also increased as a function of the pH. After multiple transfers of PS spheres from the Si wafer to the air–water interface, the surface of the water beaker was filled with grains containing hcp PS nanosphere arrays. The voids between the ordered grains were reduced by adding 2 μL of an aqueous solution of sodium dodecyl sulfate to the surface, pushing the grains together. Finally, the monolayer of PS spheres was transferred onto cleaned control MEA, which was previously treated with the O2 plasma cleaner for 3 min, by immersing the glass below the water interface and gently “fishing” the PS sphere monolayer. After the substrates were allowed to dry at an angle, hcp PS sphere arrays were formed on the control MEA and ready to be used as a colloidal mask. Then, the samples were loaded in the chamber of an electron beam evaporator vertically with respect to the source, first an adhesion layer of 2 nm of Ti followed by 200 nm of Au (pressure chamber: 2 × 10^–7^ Pa, rate: 0.2 Ȧ /s; E-beam PVD75 Kurt J. Lesker company) were deposited, without rotation. Finally, the PS spheres were removed by tape stripping, and only the arrays of Au pyramids were left on the control MEA.

### Au NP Array Morphological and Optical Characterization

The morphology of the Au nano pyramids was characterized by imaging the samples with a scanning electron microscope system (FEI Helios NanoLab 650). The transmission and reflection spectra of the Au pyramids at normal incidence in the wavelength range of 400–1000 nm were collected with an ellipsometer (J.A. Woolam Co. VVASE ellipsometer).

### Photocurrent Measurement

The 1064 nm Nd:YAG [neodymium:yttrium–aluminum–garnet] solid-state laser (Plecter Duo [Coherent]) was used as the light source, for which the emission is in ultrashort pulses at 8 ps with 80 MHz repetition rate. The pulsed beam was then switched ON and OFF at the desired pulse length, generating pulse trains ranging from microseconds to hundreds of milliseconds. Throughout the paper, the term pulse length referred to the ON time of the 8 ps pulsed laser, defined with an acousto-optic modulator (AOM) or a mechanical shutter controlled by a TTL signal from the analog-to-digital signal converters (ADC/DAC Axon Digidata 1550B plus HumSilencer) connected to the software (Axon pCLAMP). The laser was combined with an upright microscope (Ecplise FN-1 from Nikon) able to accommodate the multichannel systems acquisition system directly on the microscope stage. A 20× objective (NA 1.0) was used in order to focus the NIR laser used for the stimulation. For this purpose, the Au NP MEA electrodes were immersed in PBS and connected with the amplifier (Axopatch 200B).

### HiPSC-Derived Neurons Culture on MEAs

Human induced pluripotent stem cell (hiPSC) derived neurons (iCell GlutaNeurons) were purchased from Fujifilm Cellular Dynamics, Inc. These cells are a highly pure population of human glutamatergic cortical neurons. MEAs devices were sterilized under UV light for 30 min and coated with 0.07% polyethylenimine (Sigma-Aldrich) diluted in borate buffer for 1 h at room temperature. The devices were then washed four times with sterile water and air-dried overnight in a biological hood. GlutaNeurons were seeded at a density of 120,000 cells/well in a drop directly over the recording electrode area of MEAs and grown according to the manufacturer’s instructions. A 50% medium change was performed 1 day postplating and then every other day. Photostimulation experiments were performed starting from 28 days postplating and repeated every day.

### Optical Stimulation Procedure

For the optical stimulation protocol, we use the same optical setup presented in previous works.^37^ The photostimulation beam at 1064 nm is delivered in an upright configuration from above through a 20× objective. The beam is focused onto a single MEA electrode for each stimulation epoch; neighboring electrodes remain unilluminated and are recorded concurrently as controls. For each panel/experiment, we report the on-sample average power at the objective output. Consistent with single-electrode targeting, we did not observe stimulation on nonilluminated neighboring electrodes under the same power and timing parameters. At first, the physiological extracellular activity was recorded for about 10 min to characterize the culture. Second, the laser pulse protocol was applied on the Au pyramids and were used to induce cellular stimulation. Typically, a laser power of 2–10 mW was used.

## Supplementary Material



## Notes

Additional SEM image of Au pyramids on TiN electrode, electrochemical impedance spectroscopy of Au pyramids on TiN electrode after 3 cultures, and experimental procedure (PDF)

## References

[1] Stephan M., Volkmann P., Rossner M. J. (2019). Assessing
Behavior and Cognition
in Rodents, Nonhuman Primates, and Humans: Where Are the Limits of
Translation?. Dialogues Clin. Neurosci.

[2] Lamotte J. D. De., Roqueviere S., Gautier H., Raban E., Bouré C., Fonfria E., Krupp J., Nicoleau C. (2021). HiPSC-Derived Neurons
Provide a Robust and Physiologically Relevant In Vitro Platform to
Test Botulinum Neurotoxins. Front. Pharmacol..

[3] Zhang Q., Zeng Y., Zhang T., Yang T. (2021). Comparison Between
Human and Rodent Neurons for Persistent Activity Performance: A Biologically
Plausible Computational Investigation. Front
Syst. Neurosci.

[4] Ardhanareeswaran K., Mariani J., Coppola G., Abyzov A., Vaccarino F. M. (2017). Human Induced
Pluripotent Stem Cells for Modelling Neurodevelopmental Disorders. Nat. Rev. Neurol..

[5] Autar K., Guo X., Rumsey J. W., Long C. J., Akanda N., Jackson M., Narasimhan N. S., Caneus J., Morgan D., Hickman J. J. (2022). A Functional
HiPSC-Cortical Neuron Differentiation and Maturation Model and Its
Application to Neurological Disorders. Stem
Cell Rep..

[6] De
Luca C., Colangelo A. M., Virtuoso A., Alberghina L., Papa M. (2020). Neurons, Glia, Extracellular Matrix and Neurovascular Unit: A Systems
Biology Approach to the Complexity of Synaptic Plasticity in Health
and Disease. Int. J. Mol. Sci..

[7] Nair A., Chauhan P., Saha B., Kubatzky K. F. (2019). Conceptual Evolution
of Cell Signaling. Int. J. Mol. Sci..

[8] Zhang H., Fang H., Liu D., Zhang Y., Adu-Amankwaah J., Yuan J., Tan R., Zhu J. (2022). Applications and Challenges
of Rhodopsin-Based Optogenetics in Biomedicine. Front Neurosci.

[9] Emiliani V., Entcheva E., Hedrich R., Hegemann P., Konrad K. R., Lüscher C., Mahn M., Pan Z.-H., Sims R. R., Vierock J., Yizhar O. (2022). Optogenetics for Light Control of
Biological Systems. Nat. Rev. Methods Primers.

[10] Shen Y., Campbell R. E., Côté D.
C., Paquet M.-E. (2020). Challenges
for Therapeutic Applications of Opsin-Based Optogenetic Tools in Humans. Front Neural Circuits.

[11] Bruno G., Melle G., Barbaglia A., Iachetta G., Melikov R., Perrone M., Dipalo M., De Angelis F. (2021). All-Optical
and Label-Free Stimulation of Action Potentials in Neurons and Cardiomyocytes
by Plasmonic Porous Metamaterials. Adv. Sci..

[12] Jiang Y., Li X., Liu B., Yi J., Fang Y., Shi F., Gao X., Sudzilovsky E., Parameswaran R., Koehler K., Nair V., Yue J., Guo K., Fang Y., Tsai H.-M., Freyermuth G., Wong R. C. S., Kao C.-M., Chen C.-T., Nicholls A. W., Wu X., Shepherd G. M. G., Tian B. (2018). Rational Design of
Silicon Structures for Optically Controlled Multiscale Biointerfaces. Nat. Biomed. Eng..

[13] Roth R. H., Ding J. B. (2020). From Neurons to
Cognition: Technologies for Precise
Recording of Neural Activity Underlying Behavior. BME Front..

[14] Xu S., Momin M., Ahmed S., Hossain A., Veeramuthu L., Pandiyan A., Kuo C., Zhou T. (2023). Illuminating the Brain:
Advances and Perspectives in Optoelectronics for Neural Activity Monitoring
and Modulation. Adv. Mater..

[15] Delbeke J., Hoffman L., Mols K., Braeken D., Prodanov D. (2017). And Then There
Was Light: Perspectives of Optogenetics for Deep Brain Stimulation
and Neuromodulation. Front. Neurosci..

[16] Eder M., Zieglgänsberger W., Dodt H.-U. (2004). Shining Light on
Neurons - Elucidation of Neuronal Functions by Photostimulation. Rev. Neurosci.

[17] Callaway E. M., Yuste R. (2002). Stimulating Neurons with Light. Curr. Opin.
Neurobiol..

[18] Zilio P., Dipalo M., Tantussi F., Messina G. C., de Angelis F. (2017). Hot Electrons
in Water: Injection and Ponderomotive Acceleration by Means of Plasmonic
Nanoelectrodes. Light Sci. Appl..

[19] Dipalo M., Amin H., Lovato L., Moia F., Caprettini V., Messina G. C., Tantussi F., Berdondini L., De Angelis F. (2017). Intracellular and Extracellular Recording
of Spontaneous
Action Potentials in Mammalian Neurons and Cardiac Cells with 3D Plasmonic
Nanoelectrodes. Nano Lett..

[20] Dipalo M., Melle G., Lovato L., Jacassi A., Santoro F., Caprettini V., Schirato A., Alabastri A., Garoli D., Bruno G., Tantussi F., De Angelis F. (2018). Plasmonic
Meta-Electrodes Allow Intracellular Recordings at Network Level on
High-Density CMOS-Multi-Electrode Arrays. Nat.
Nanotechnol..

[21] Garoli D., Calandrini E., Giovannini G., Hubarevich A., Caligiuri V., De Angelis F. (2019). Nanoporous
Gold Metamaterials for
High Sensitivity Plasmonic Sensing. Nanoscale
Horiz..

[22] Melle G., Bruno G., Maccaferri N., Iachetta G., Colistra N., Barbaglia A., Dipalo M., De Angelis F. (2020). Intracellular
Recording of Human Cardiac Action Potentials on Market-Available Multielectrode
Array Platforms. Front. Bioeng. Biotechnol..

[23] Bruno G., Melle G., Barbaglia A., Iachetta G., Melikov R., Perrone M., Dipalo M., De Angelis F. (2021). All-Optical
and Label-Free Stimulation of Action Potentials in Neurons and Cardiomyocytes
by Plasmonic Porous Metamaterials. Adv. Sci..

[24] Iarossi M., Hubarevich A., Iachetta G., Dipalo M., Huang J.-A., Darvill D., De Angelis F. (2022). Probing ND7/23
Neuronal Cells before
and after Differentiation with SERS Using Sharp-Tipped Au Nanopyramid
Arrays. Sens Actuators B Chem..

[25] Carvalho-de-Souza J.
L., Treger J. S., Dang B., Kent S. B. H., Pepperberg D. R., Bezanilla F. (2015). Photosensitivity of Neurons Enabled by Cell-Targeted
Gold Nanoparticles. Neuron.

[26] Chapman C. A. R., Chen H., Stamou M., Biener J., Biener M. M., Lein P. J., Seker E. (2015). Nanoporous
Gold as a Neural Interface
Coating: Effects of Topography, Surface Chemistry, and Feature Size. ACS Appl. Mater. Interfaces.

[27] Gerwig R., Fuchsberger K., Schroeppel B., Link G. S., Heusel G., Kraushaar U., Schuhmann W., Stett A., Stelzle M. (2012). PEDOT–CNT
Composite Microelectrodes for Recording and Electrostimulation Applications:
Fabrication, Morphology, and Electrical Properties. Front. Neuroeng..

[28] Melikov R., Srivastava S. B., Karatum O., Dogru-Yuksel I. B., Bahmani Jalali H., Sadeghi S., Dikbas U. M., Ulgut B., Kavakli I. H., Cetin A. E., Nizamoglu S. (2020). Plasmon-Coupled
Photocapacitor Neuromodulators. ACS Appl. Mater.
Interfaces.

[29] Zheng X. S., Tan C., Castagnola E., Cui X. T. (2021). Electrode Materials for Chronic Electrical
Microstimulation. Adv. Healthcare Mater..

[30] Schmidt T., Jakešová M., Đerek V., Kornmueller K., Tiapko O., Bischof H., Burgstaller S., Waldherr L., Nowakowska M., Baumgartner C., Üçal M., Leitinger G., Scheruebel S., Patz S., Malli R., Głowacki E. D., Rienmüller T., Schindl R. (2022). Light Stimulation of Neurons on Organic
Photocapacitors Induces Action Potentials with Millisecond Precision. Adv. Mater. Technol..

[31] Zhao Y., Iarossi M., Maccaferri N. (2023). Hyperbolic metamaterial
nanoparticles random array for thermoplasmonics in the II and III
near-infrared windows. Appl. Phys. Lett..

[32] Carvalho-de-Souza J.
L., Treger J. S., Dang B., Kent S. B. H., Pepperberg D. R., Bezanilla F. (2015). Photosensitivity of Neurons Enabled by Cell-Targeted
Gold Nanoparticles. Neuron.

[33] Alahi M. E. E., Rizu M. I., Tina F. W., Huang Z., Nag A., Afsarimanesh N. (2023). Recent Advancements
in Graphene-Based Implantable Electrodes
for Neural Recording/Stimulation. Sensors.

[34] Darvill D., Iarossi M., Abraham
Ekeroth R. M., Hubarevich A., Huang J.-A., De Angelis F. (2021). Breaking the
Symmetry of Nanosphere
Lithography with Anisotropic Plasma Etching Induced by Temperature
Gradients. Nanoscale Adv..

[35] Vogel N., Weiss C. K., Landfester K. (2012). From Soft
to Hard: The Generation
of Functional and Complex Colloidal Monolayers for Nanolithography. Soft Matter.

[36] Vogel N., Goerres S., Landfester K., Weiss C. K. (2011). A Convenient Method
to Produce Close- and Non-close-Packed Monolayers Using Direct Assembly
at the Air–Water Interface and Subsequent Plasma-Induced Size
Reduction. Macromol. Chem. Phys..

[37] Melikov R., Iachetta G., d’Amora M., Melle G., Conti S., Tantussi F., Dipalo M., De Angelis F. (2025). Longitudinal
and Noninvasive Intracellular Recordings of Spontaneous Electrophysiological
Activity in Rat Primary Neurons on Planar MEA Electrodes. Adv. Mater..

