# Supplementary material for: Nongenetic Photostimulation of hiPSC Neurons Using Plasmonic Nanopyramids
Source: ACS Photonics. 2025 Oct 27;12(11):6237–44. doi: 10.1021/acsphotonics.5c01708 (PMC12636067; doi:10.1021/acsphotonics.5c01708)
Supplement: Supplementary file 1 [file ph5c01708_si_001.pdf]

Supporting Information

Non-genetic photostimulation of hiPSC neurons using plasmonic nanopyramids

Rustamzhon Melikov[a,+], *Giuseppina Iachetta* [a,+], *Marzia Iarossi*[b], *Marta d'Amora* [a,c], ,

Christian Tentellino[a], *Julien Maxime Hurtaud*[a], *Francesco Tantussi*[a], *Michele Dipalo*[a],

Francesco De Angelis[a*]

[a] Italian Institute of Technology, Genoa, 16162, Italy
[b] Department of Biomedical Engineering, Technion - Israel Institute of Technology, 32000 Haifa, Israel
[c] Department of Biology, University of Pisa, 56127 Pisa, Italy
[+] equal contribution
email: (francesco.deangelis@iit.it)

Keywords
electrophysiology, photostimulation, micro electrode arrays, plasmonics, locilized surface plasmon resonance, neurons, Human induced pluripotent stem cell, capacitive photocurrent.

Table of Contents

SEM image of Au Pyramids on TiN electrode **2**

EIS of Au pyramid electrodes after three cultures **2**

Experimental Procedure **3**

REFERENCES **6**

SEM image of Au Pyramids on TiN electrode

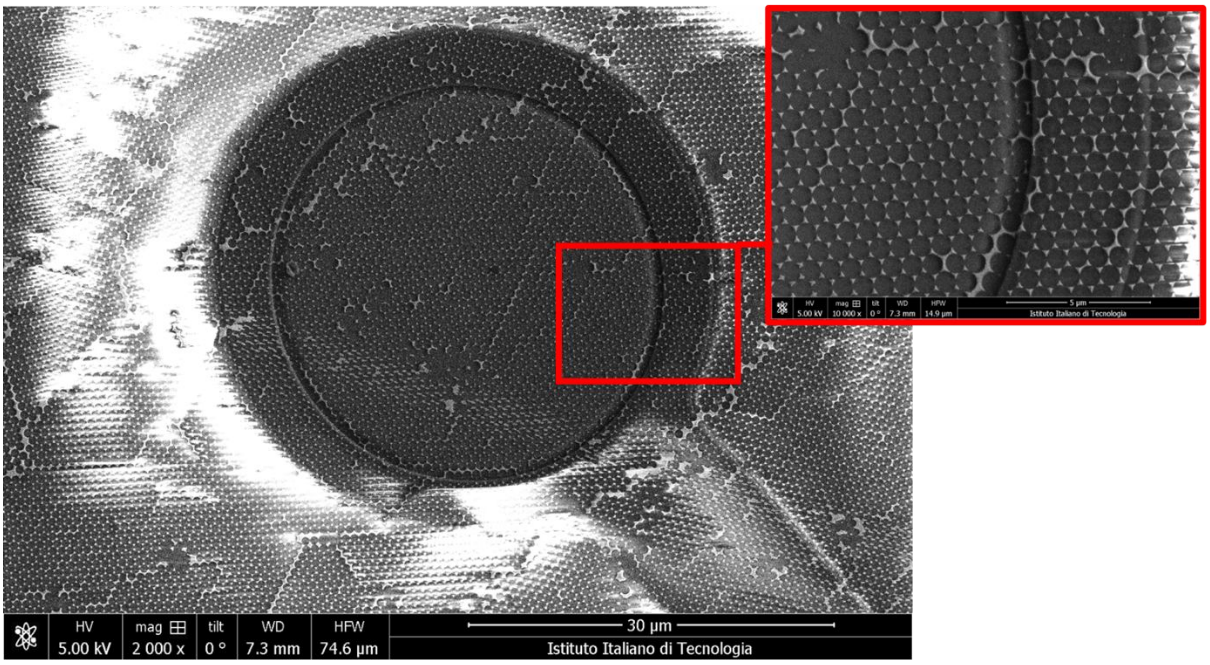

Figure S1. *SEM images of Au pyramids on TiN electrode with scale bars of 30 and 5 µm respectively on left and right. The density of pyramid is 4* per µm².

EIS of Au pyramid electrodes after three cultures

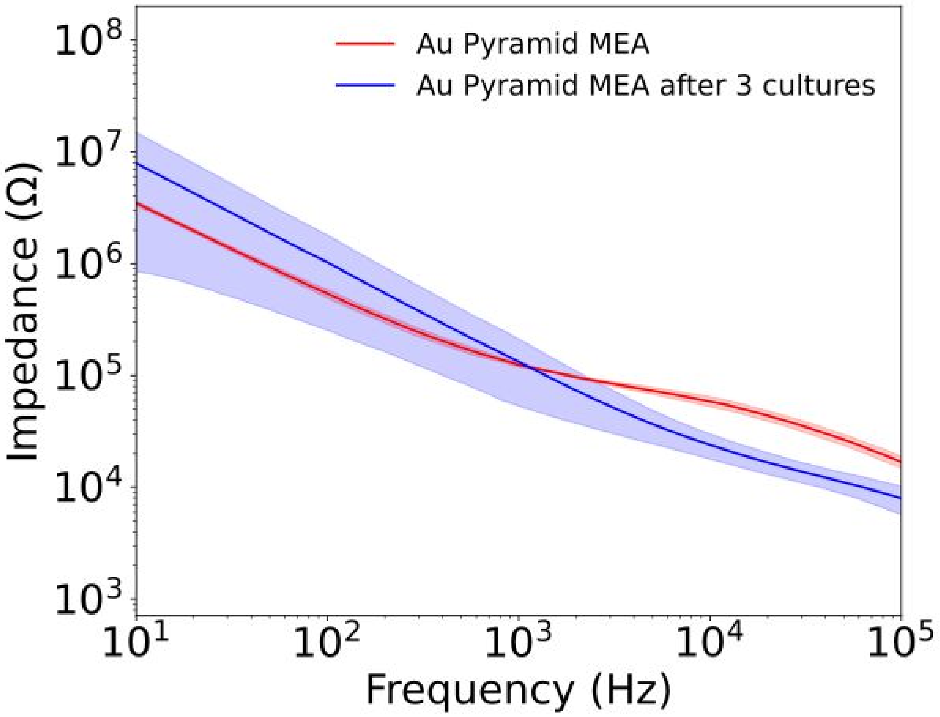

Figure S2. Electrochemical impedance spectroscopy of Au pyramid MEA after three cultures

Experimental Procedure

Fabrication of Au pyramids: The fabrication route to Au NP arrays is based on colloidal lithography. Firstly, negatively charged polystyrene (PS) spheres (purchased from microParticles GmbH) with superficial carboxylic acid groups and an average diameter of 750 nm were assembled in a hexagonally-close-packed (hcp) monolayer. This was achieved by using an interfacial air-water self-assembly technique[1,2]. Briefly, the aqueous solution of PS spheres (5 wt%) was diluted in a 1:1 ratio in ethanol and then pipetted onto a Si wafer suspended at an angle of 80◦ with its bottom edge in contact with a water bath. The Si wafer was first treated with an oxygen (O2) plasma cleaner (power: 100 W, flow rate: 25 sccm; Gambetti Plasma Cleaner System) for 10 min to make the surface completely hydrophilic, to encourage spreading. In this way, the excess PS spheres could flow away in the water bath whilst ordered hcp monolayers were formed on the Si water after the solvent evaporates. A second plastic beaker was filled with deionized water and the pH was increased up to 9 by adding a solution of sodium hydroxide. The pH of the water bath played an important role on the subsequent step in which the Si wafer was gently dropped in the water bath to transfer the PS spheres monolayers at the water-air interface of the second bath. The pH of the water bath affected the repulsion between neighbouring PS spheres because at higher pH values the surface charge density on each particle increased as a result of the deprotonation of the carboxylic acid groups[33]. By increasing the particles' electrostatic repulsion, the length of the triangular interstices formed by neighbouring spheres also increased as a function of the pH. After multiple transfers of PS spheres from the Si wafer to the air-water interface, the surface of the water beaker was filled with grains containing hcp PS nanosphere arrays. The voids between the ordered grains were reduced by adding 2 μL of an aqueous solution of sodium

dodecyl sulfate to the surface, pushing the grains together. Finally, the monolayer of PS spheres was transferred onto cleaned control MEA, which were previously treated with the O2 plasma cleaner for 3 min, by immersing the glass below the water interface and gently “fishing” the PS sphere monolayer. After allowing the substrates to dry at an angle, hcp PS sphere arrays were formed on the control MEA and ready to be used as a colloidal mask. Then, the samples were loaded in the chamber of an electron beam evaporator vertically with respect to the source, firstly an adhesion layer of 2 nm of Ti followed by 200 nm of Au (pressure chamber: 2 · 10-7 Pa, rate: 0.2 A˙ /s; E-beam PVD75 Kurt J. Lesker company) were deposited, without rotation. Finally, the PS spheres were removed by tape stripping and only the arrays of Au pyramids were left on the control MEA.

Au NP Array morphological and optical characterization: The morphology of the Au nano pyramids was characterized by imaging the samples with a scanning electron microscope system (FEI Helios NanoLab 650). The transmission and reflection spectra of the Au pyramids at normal incidence in the wavelength range 400–1000 nm were collected with an ellipsometer (J.A. Woolam Co. VVASE ellipsometer).

Photocurrent Measurement: The 1064 nm (Nd:YAG [neodymium:yttrium–aluminum–garnet] solid-state laser (Plecter Duo [Coherent]) was used as the light source, for which the emission is in ultra-short pulses at 8 ps with 80 MHz repetition rate. The pulsed beam was then switched ON and OFF at the desired pulse length, generating pulse trains ranging from microseconds to hundreds of milliseconds. Throughout the manuscript, the term pulse length referred to the ON time of the 8 ps pulsed laser, defined with an acousto-optic modulator (AOM) or a mechanical shutter controlled by a TTL signal from the analogto-digital signal converters (ADC/DAC Axon Digidata 1550B plus HumSilencer) connected to the software

(Axon pCLAMP). The laser was combined to an upright microscope (Ecplise FN-1 from Nikon) able to accommodate the Multichannel systems acquisition system directly on the microscope stage. A 20 × objective (NA 1.0) was used in order to focus the NIR laser used for stimulation. For the purpose, the Au pyramid MEA electrodes were immersed in PBS without neurons and connected with the amplifier (Axopatch 200B).

HiPSC-derived neurons culture on MEAs: Human induced pluripotent stem cells (hiPSC) derived neurons (iCell® GlutaNeurons) were purchased from Fujifilm Cellular Dynamics, Inc. These cells are a highly pure population of human glutamatergic cortical neurons. MEAs devices were sterilized under UV light for 30 minutes and coated with 0.07% polyethyleneimine (Sigma-Aldrich) diluted in borate buffer for 1 h at room temperature. The devices were then washed four times with sterile water and air-dried overnight in a biological hood. GlutaNeurons were seeded at a density of 120,000 cells/well in a drop directly over the recording electrode area of MEAs and grown according to the manufacturer's instructions. A 50% medium change was performed one day post-plating and then every other day. Photostimulation experiments were performed starting from 28 days post-plating and repeated every day.

Optical Stimulation Procedure: For the optical stimulation protocol, we use the same optical setup presented in previous works[3], The photostimulation beam 1064nm is delivered in an upright configuration **from above through a 20× objective**. The beam is **focused onto a single MEA electrode** for each stimulation epoch; neighboring electrodes remain unilluminated and are recorded concurrently as controls. For each panel/experiment we report the **on-sample average power** at the objective output. Consistent with single-electrode targeting, we did not observe stimulation on non-illuminated neighboring electrodes under the same power and timing parameters. At first, the physiological extracellular activity was recorded for about 10

min to characterize the culture. Second, laser pulse protocol was applied on the Au pyramids and were used to induce cellular stimulation. Typically, a laser power of 2 - 10 mW was used.

REFERENCES

(1) Darvill, D.; Iarossi, M.; Abraham Ekeroth, R. M.; Hubarevich, A.; Huang, J.-A.; De Angelis, F. Breaking the Symmetry of Nanosphere Lithography with Anisotropic Plasma Etching Induced by Temperature Gradients. *Nanoscale Adv* **2021**, *3* (2), 359–369. https://doi.org/10.1039/D0NA00718H.

(2) Vogel, N.; Goerres, S.; Landfester, K.; Weiss, C. K. A Convenient Method to Produce Close- and Non-close-Packed Monolayers Using Direct Assembly at the Air–Water Interface and Subsequent Plasma-Induced Size Reduction. *Macromol Chem Phys* **2011**, *212* (16), 1719–1734. https://doi.org/10.1002/macp.201100187.

(3) Melikov, R.; Iachetta, G.; d'Amora, M.; Melle, G.; Conti, S.; Tantussi, F.; Dipalo, M.; De Angelis, F. Longitudinal and Noninvasive Intracellular Recordings of Spontaneous Electrophysiological Activity in Rat Primary Neurons on Planar MEA Electrodes. *Advanced Materials* **2025**. https://doi.org/10.1002/adma.202412697.
